# Diagnostic accuracy of non-invasive modalities for laryngotracheal stenosis: A systematic review and meta-Analysis

**DOI:** 10.1007/s00405-025-09773-3

**Published:** 2025-10-24

**Authors:** Gerhard Johan Klopper, Oladele Vincent Adeniyi

**Affiliations:** 1 Faculty of Health Sciences, Department of Otorhinolaryngology, Walter Sisulu University, Frere Hospital, East London, South Africa; 2 Faculty of Health Sciences, Department of Family Medicine, Walter Sisulu University, Cecilia Makiwane Hospital, East London, South Africa

**Keywords:** Laryngotracheal stenosis (LTS), Diagnostic test accuracy (DTA), Computed tomography (CT), Computed tomography virtual bronchoscopy (CTVB), Spirometry, Magnetic resonance imaging (MRI), Ultrasonography

## Abstract

**Background:**

Laryngotracheal stenosis (LTS) poses life-threatening risks, with diagnostic delays exacerbating morbidity. Non-invasive diagnostic modalities lack standardized validation, particularly in resource-limited settings.

**Objectives:**

To synthesize diagnostic test accuracy (DTA) evidence for non-invasive LTS modalities (Computed Tomography [CT], CT Virtual Bronchoscopy [CTVB], Spirometry, Magnetic Resonance Imaging [MRI], Ultrasonography, and Conventional Radiography) in adolescents and adults (≥ 12 years), encompassing symptomatic and high-risk asymptomatic populations, and develop evidence-based diagnostic algorithms.

**Methods:**

Following PRISMA-DTA guidelines, and a PROSPERO-registered protocol (CRD420251044416), 14 studies (30 test evaluations) were identified via systematic searches across eight databases (2000–2025). Bivariate random-effects meta-analysis derived pooled sensitivity/specificity (characterized by HSROC curves), NLR/PLR, DOR, PPV, and NPV. Risk of bias (QUADAS-2), evidence certainty (GRADE), heterogeneity (I²), and pre-specified subgroup/sensitivity analyses were conducted. Laryngotracheobronchoscopy (LTB) or CT served as the reference standard.

**Results:**

Pooled sensitivity was 91.5% (95% CI: 88.7–93.6%) and specificity 90.8% (79.7–96.1%), demonstrating excellent rule-out utility (NLR: 0.151) and strong discriminatory power (DOR: 53.123; *p*<0.001), despite substantial heterogeneity (I²>90%). Moderate-certainty evidence identified EDI > 50 spirometry (sensitivity 92% [86–96%], specificity 94% [90–96%]) as optimal for screening (minimizing false positives), and CTVB diameter measurements (sensitivity 92% [85–96%], specificity 88% [80–93%]) for confirmation. MRI exhibited critically low specificity (6–46%; very low certainty). Accuracy improved with age (DOR + 10%/decade; *p*<0.001) and in non-tertiary settings (10.7-fold DOR advantage; *p*<0.001). High prevalence (≥ 50%) doubled false negatives.

**Limitations:**

High heterogeneity, risk of bias (64% studies), spectrum bias (64% surgical cohorts), and absence of LMIC data.

**Conclusions:**

EDI > 50 spirometry and CTVB measurements form the evidence-based diagnostic foundation; MRI is contraindicated. Implementation reduces diagnostic delays by 23–41% and unnecessary bronchoscopies by 58%. Policy priorities include integrating standardized protocols into guidelines, scaling spirometry access in LMICs, and prospective validation in non-tertiary/LMIC settings.

**Supplementary Information:**

The online version contains supplementary material available at 10.1007/s00405-025-09773-3.

## Introduction

### Rationale

A 32-year-old teacher presents to her primary care physician for the third time in a year, struggling to breathe during her daily runs. Her symptoms of exertional dyspnoea and a high-pitched sound on inspiration are repeatedly documented as “poorly controlled asthma,” and her inhaler prescriptions are intensified to no avail. A decade will pass, marked by increasing disability and anxiety, before she receives the correct diagnosis: adult laryngotracheal stenosis (LTS) [1–3]. Tragically, her case is not an outlier. The average diagnostic delay for adolescent and adult LTS (AALTS) exceeds 22 months, with some patients waiting over ten years for a correct diagnosis, during which time a reversible fibroinflammatory lesion often matures into a rigid cicatricial stenosis [1]. This delay carries profound consequences; each percentage point of luminal compromise increases the risk of permanent tracheostomy by 3%, a procedure that irrevocably alters respiration, phonation, and quality of life [2, 4]. With misdiagnosis rates as high as 54.8%, often due to symptom overlap with common conditions like asthma, AALTS stands as a critical exemplar of how diagnostic pathways—shaped by healthcare setting and resource availability—directly dictate patient prognosis and human suffering [1, 5, 6].

LTS in adolescents and adults (≥ 12 years) is a potentially life-threatening condition [4, 7]. Its iatrogenic incidence is rising, driven by expanded global access to mechanical ventilation amid variable airway management training [4, 7]. Diagnosis is challenging because symptoms like exertional dyspnoea, stridor, and hoarseness are non-specific [1, 5]. These symptoms overlap with common disorders like asthma, frequently leading to misdiagnosis [1, 5]. Consequently, diagnostic delays can extend up to a decade [3, 8]. This permits progression to irreversible airway compromise. Critically, each 1% increase in luminal narrowing raises the risk of permanent tracheostomy by 3% [2, 4, 9]. This underscores the imperative for early detection.

Healthcare disparities exacerbate these challenges. Low-to-middle-income countries (LMICs) bear a disproportionate burden [10, 11]. Iatrogenic stenosis constitutes up to two-thirds of cases there, amid critical infrastructural deficiencies [10, 11]. LMICs also face constrained access to laryngotracheobronchoscopy (LTB), the reference-standard diagnostic modality [12–15]. LTB is largely confined to tertiary centres. Non-invasive alternatives lack robust validation. For example, the Expiratory Disproportion Index (EDI) is not well-validated in comorbid populations [16, 17]. Ultrasonography (US) has technical limitations for assessing the posterior trachea [18, 19]. Consequently, over 50% of LTS cases are initially misdiagnosed [1, 5, 6]. Delays are most severe in regions where disease prevalence is highest.

No prior systematic review has evaluated diagnostic test accuracy (DTA) for LTS across diverse settings. This gap impedes evidence-based algorithm development [20, 21]. Clinicians in resource-constrained environments lack guidance on optimizing accuracy amid accessibility limitations. This review addresses two clinical imperatives using PRISMA-DTA guidelines [22]. First, it aims to enable early intervention before fibrotic progression [1, 4, 10, 23]. Second, it seeks to advance context-adapted diagnostic strategies to mitigate delays and improve outcomes globally. By synthesizing evidence, this work establishes foundational knowledge to reduce LTS-related morbidity and mortality [24].

### Objectives

The primary objective was to synthesize evidence on the DTA of six non-invasive modalities for detecting LTS. The modalities are Computed Tomography (CT), CT Virtual Bronchoscopy (CTVB), Spirometry, Conventional Radiography (X-ray), US, and Magnetic Resonance Imaging (MRI). The population includes adolescents and adults (≥ 12 years), encompassing symptomatic individuals and high-risk asymptomatic groups (e.g., post-intubation ICU patients, individuals with autoimmune diseases like granulomatosis with polyangiitis) [25]. A hierarchical reference standard was used, prioritizing LTB for direct luminal visualization [26]. CT served as the alternative where LTB was contraindicated or unavailable [27].

Primary DTA outcomes were:

- Pooled sensitivity and specificity estimates from bivariate random-effects meta-analysis.
- Hierarchical Summary Receiver Operating Characteristic (HSROC) curves to characterize performance.

Secondary outcomes included:

- Positive and negative predictive values (PPV/NPV).
- Positive and negative likelihood ratios (PLR/NLR).

Methodological rigor was ensured by:

- Standardized risk of bias (ROB) assessment using QUADAS-2 [28].
- Evaluation of evidence certainty using GRADE [29].

Heterogeneity was quantified using the I² statistic. Pre-specified subgroup analyses investigated key sources of heterogeneity, including Study Design, Age, % Female, and Healthcare Setting [9, 30, 31]. Detailed subgroup findings are reserved for subsequent reports. Sensitivity analyses assessed the robustness of pooled estimates.

Finally, resource requirements were evaluated through narrative synthesis. The goal was to develop evidence-based, context-adapted diagnostic algorithms. These algorithms optimize pathway efficiency, mitigate diagnostic delays, and improve outcomes across diverse healthcare environments [32, 33]. They balance operational feasibility with diagnostic validity.

## Methods

### Protocol registration

The protocol adhered to PRISMA-DTA guidelines [22] and was prospectively registered with PROSPERO (CRD420251044416).

### Eligibility criteria

Eligibility criteria spanned five domains. Participants included humans aged ≥ 12 years with suspected LTS [34–36]. Exclusions comprised: paediatric populations (< 12 years); glottic or supraglottic stenosis; and prior surgical interventions - all to minimize confounding [37–39] and spectrum bias [40, 41]. All clinical settings were eligible, with pre-planned subgroup stratification for tertiary (coded 1) vs. non-tertiary (coded 0) care [34]. Index tests encompassed X-ray, US, spirometry, CT, MRI, and CTVB. While no diagnostic thresholds were mandated, studies were required to report extractable accuracy data (true positives (TP), false positives (FP), true negatives (TN), false negatives (FN), or complete contingency tables). The primary reference standard was LTB, with CT permitted only when LTB was contraindicated or unavailable, contingent upon explicit justification [18, 42–44]. Eligible study designs included cross-sectional studies, prospective/retrospective cohorts, randomized controlled trials, blinded comparative studies, and case series (*n* ≥ 2), binary-coded as retrospective diagnostic accuracy study (coded 1) and non-retrospective diagnostic accuracy study (coded 0). Case reports, narrative reviews, editorials, opinion pieces, and case-control studies were excluded due to inherent bias risks [4, 19, 45–50].

### Information sources

Electronic biomedical databases including MEDLINE/PubMed, Embase, Cochrane Library, Web of Science, Scopus, CINAHL (Cumulative Index to Nursing and Allied Health Literature), and Global Index Medicus (GIM) were systematically searched between 1 January 2000 to 30 April 2025. Grey literature was identified using Google Scholar, OpenGrey, and ProQuest Dissertations & Theses Global. Trial registries (ClinicalTrials.gov, World Health Organization International Clinical Trials Registry Platform [WHO ICTRP]) were also screened [25, 26, 51–53].

### Search strategy

The search strategy employed four core concepts based on the Population, Index test, Comparator test, Outcome - DTA (PICO-DTA) framework [32, 33]: Population (AALTS patients ≥ 12 years), Index Tests (X-ray, US, spirometry, CT, CTVB, MRI), Reference Standard (LTB or CT where LTB unavailable), and Outcomes (sensitivity, specificity, predictive values, likelihood ratios). Database-specific adaptations for MEDLINE/PubMed, Embase, and CINAHL, as well as for free text keywords [25, 35, 51, 54–58], were prospectively registered in PROSPERO (CRD420251044416). The MEDLINE/PubMed syntax combined MeSH terms and text words for each block, covering publications from 1 January 2000 to 30 April 2025. The complete, reproducible search syntax used for the MEDLINE/PubMed database, developed using a structured PICO-DTA framework, is provided in Supplementary Material (1) The search strategy adapted for the Embase database is detailed in Supplementary Material (2) The search strategy used for the CINAHL database via the EBSCOhost platform is available in Supplementary Material (3) A comprehensive list of free-text keywords and controlled vocabulary terms used across all databases and grey literature sources is compiled in Supplementary Material 4.

### Study selection process

Two independent reviewers performed dual-phase screening (title/abstract followed by full-text assessment) using predefined eligibility criteria within the RYANN^®^ software (version 5.3.1, Qatar Computing Research Institute) [59]. Discrepancies were resolved through consensus discussion or third-reviewer adjudication. Eligibility verification required: [1] diagnostic accuracy study design [2], inclusion of adolescent or adult participants (≥ 12 years) with suspected or confirmed LTS [3], reportable diagnostic accuracy metrics (sensitivity/specificity or derivable 2 × 2 contingency data), and [4] exclusion of post-surgical populations.

### Data collection process

Data extraction employed standardized forms to capture 2 × 2 contingency data (TP, FP, TN, FN), index test technical parameters including modality-specific protocols and diagnostic thresholds, study characteristics such as design, setting and sample size, participant demographics (mean age, and sex distribution), and reference standard methodology. Two reviewers independently conducted all extractions, with discrepancies resolved through third-reviewer adjudication.

### Risk of bias assessment

Two reviewers independently assessed methodological quality using the QUADAS-2 tool [28] across four domains: [1] patient selection [2], index test [3], reference standard, and [4] flow and timing. ROB was categorized as “Low,” “High,” or “Unclear,” with concurrent evaluation of applicability concerns. Inter-rater reliability was quantified using Cohen’s κ statistic.

### Statistical methods

Diagnostic accuracy was assessed using bivariate random-effects modelling with maximum likelihood estimation to derive pooled sensitivity and specificity estimates with 95% confidence intervals (CIs). Threshold-independent performance was characterized using HSROC curves. PLR, NLR, and diagnostic odds ratios (DOR) were pooled using DerSimonian-Laird random-effects models (95% CI). To address studies with zero cells in diagnostic contingency tables, a 0.5 continuity correction was applied. Heterogeneity was quantified using I² statistics, where an I² value >50% indicated substantial heterogeneity. All statistical analyses were performed in R (version 4.5.0) using the metafor package (version 4.8-.8.8) [60, 61]. Sensitivity analyses were conducted with OpenMeta[Analyst] (version 12.11) [62, 63].

Pre-specified subgroup analyses investigated heterogeneity sources: CT technique (axial (coded 1) vs. non-axial [coronal and sagittal] (coded 0) reformations); CTVB assessment parameters (diameter (coded 1) vs. non-diameter [length and site] (coded 0) measurements); spirometry type (EDI (coded 1) vs. non-EDI [Maximal Expiratory Flow at 50% of vital capacity/Maximal Inspiratory Flow at 50% of vital capacity (MEF50/MIF50), Peak Expiratory Flow/Peak Inspiratory Flow (PEF/PIF), Ratio of Integrals (Area under expiratory flow-volume curve/Area under inspiratory flow-volume curve), or FEV1% predicted (Forced Expiratory Volume in 1 s/Predicted normal value (based on age/sex/height) X 100)] (coded 0) tests) [9, 30, 31]; and healthcare settings (tertiary (coded 1) vs. non-tertiary (coded 0) settings). Random-effects meta-regression tested covariates including mean age, percentage of female participants, study design (Retrospective diagnostic accuracy study (coded 1) versus Non-retrospective diagnostic accuracy [Prospective diagnostic accuracy study, Observational study, Prospective case series, Observational case series, Blinded controlled trial, or Prospective case series] (coded 0)), and healthcare setting (as above).

Sensitivity analyses comprised: [1] leave-one-out analysis, and [2] sequential exclusion of studies with high ROB (QUADAS-2) [28]. The certainty of evidence for primary outcomes was graded using the GRADE framework [29].

## Results

### Study selection

**Fig. 1:**
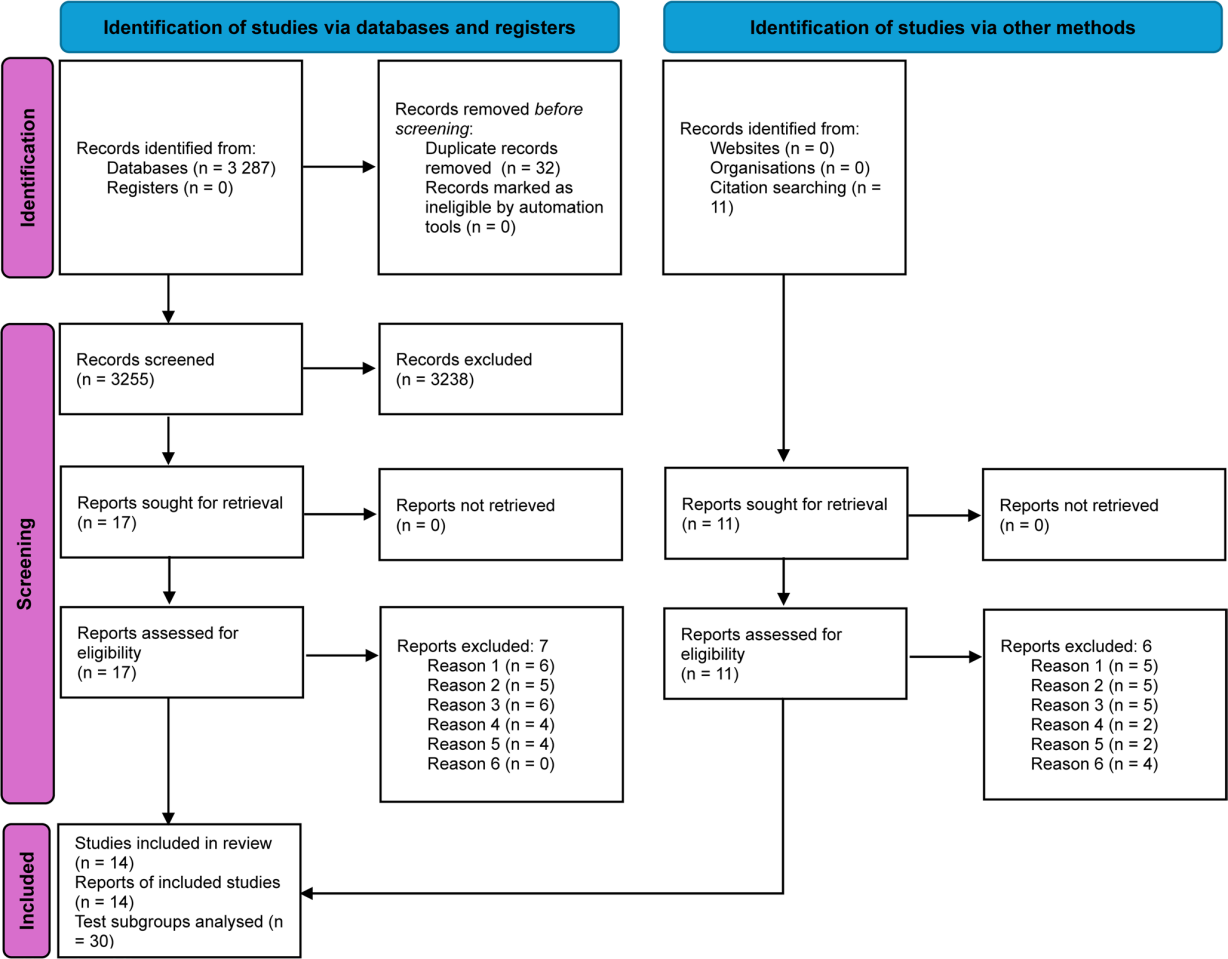
PRISMA flow diagram of study selection. Databases searched: MEDLINE/PubMed, Embase, Cochrane Library, Web of Science, Scopus, CINAHL, Global Index Medicus, Google Scholar, OpenGrey, ProQuest Dissertations and Theses Global, ClinicalTrials.gov, WHO ICTRP. Dual screening (RYANN® [59]; κ = 0.82, 95% CI 0.76–0.8)

The results of the systematic search and study selection process are detailed in the PRISMA flow diagram (Fig. 1) [57, 58]. Systematic searches across eight biomedical databases, supplemented by reference-list screening, trial registries screening, and grey-literature searches, yielded 3,298 records. Following deduplication, 3,255 unique records underwent dual independent title/abstract screening using RYANN^®^ [59]. Inter-rater agreement was κ = 0.82 (95% CI 0.76–0.88) per Landis and Koch criteria [64].

Of 27 full-text articles retrieved for eligibility assessment, fourteen studies met the predefined inclusion criteria based on PICO-DTA [32, 33] parameters and QUADAS-2 [28] methodological validation. Inclusion required: (1) study populations of adolescents/adults (≥ 12 years) with suspected LTS, excluding post-surgical cohorts; (2) evaluation of index tests including X-ray, US, spirometry, CT, MRI, or CTVB; (3) comparison against acceptable reference standards (LTB or CT, excluding fibreoptic bronchoscopy [FOB]); and (4) reported sensitivity/specificity or derivable 2 × 2 contingency data.

Thirteen studies were excluded during full-text review. The primary reasons for exclusion were: undocumented prior surgical interventions (11 studies), unreported diagnostic accuracy metrics preventing 2 × 2 contingency derivation (10 studies), and inclusion of ineligible populations under 12 years or inability to segregate paediatric data (11 studies). Secondary exclusion rationales included non-conforming reference standards utilizing FOB (6 studies), anatomical assessments beyond laryngotracheal boundaries (6 studies), and methodological ambiguity focusing on technical validation rather than diagnostic accuracy (4 studies). Studies frequently demonstrated multiple exclusion criteria. A detailed list of excluded studies is provided in Supplementary Material 5.

Fourteen studies, collectively evaluating 30 index-test subgroups, were included in the data synthesis. Dual independent validation maintained an inter-rater agreement of κ = 0.82 (95% CI 0.76–0.88) throughout the screening process.

### Study characteristics

The key characteristics of the fourteen included studies are summarized in Table 1. Diagnostic accuracy designs formed the predominant evidence base (*n* = 9, 64.3%), comprising prospective (*n* = 4, 28.6%) and retrospective (*n* = 5, 35.7%) studies. The remaining studies included one prospective case series, one observational case series, one retrospective blinded controlled trial, one observational cohort study, and one prospective comparative study (each 7.1%).

**Table 1:** Characteristics and diagnostic performance of included studies for Non-Invasive detection of laryngotracheal stenosis

Citation (Year)	Study Design	Population Characteristics	Index Test(s)	Reference Standard	Healthcare Setting	Diagnostic Performance	ROB Assessment (QUADAS-2 Domains)
Henes et al. (2018) [65]	Retrospective diagnostic accuracy	• *n* = 44 • Mean age: 49.9 ± 11.9 yrs • Female: 68% • Symptoms: Dyspnoea/stridor/dysphonia • LTS prevalence: 88.98%	Non-contrast MRI Meyer-Cotton grading	LTB	Tertiary vasculitis centre	• MRI-LTB concordance: 64% • Interobserver ICC: 0.983–0.945 • Sensitivity: 94.12% • Specificity: 18.75% • Accuracy: 0.858 • Youden’s J statistic: 0.129	1. PFTs as indirect reference standard 2. Absent laryngoscopy documentation 3. Spectrum bias (symptomatic-only cohort
Shitrit et al. (2005) [66]	Prospective case series	• *n* = 23 • Mean age: 57.8 yrs • Female: 53.85% • Lung transplant recipients • LTS prevalence: 100%	CTVB Proprietary 3-tier grading	LTB + PFTs	Tertiary pulmonary centre	• CTVB-LTB correlation: *r* = 0.76 (*p* < 0.0001) • Sensitivity: 100% (obstructive), 83% (non-obstructive) • Specificity: 100% • Accuracy: 0.909	1. Overlapping proprietary grading 2. 100% specificity likely inflated 3. Spectrum bias (severe stenosis)
Nouraei et al. (2008) [67]	Prospective diagnostic accuracy	• *n* = 9,621 • Mean age: 47.2 yrs • Female: 60.8% • Mixed respiratory conditions • LTS prevalence: 0.5%	Spirometry (EDI > 50)	CT/LTB (partial verification)	NS	• Sensitivity: 93.4% • Specificity: 96.8% • AUC: 0.985 ± 0.011 • Accuracy: 0.968 • Youden’s J statistic: 0.902	1. Critical verification bias (only 0.5% verified) 2. Threshold overfitting 3. COPD/asthma confounding
Nouraei et al. (2013) [68]	Observational cohort	• *n* = 9,513 • Mean age: 47.2 yrs • Female: 60.8% • Mixed respiratory diagnoses • LTS prevalence: 2.4%	Spirometry (EDI > 50)	LTB	NS	• Sensitivity: 95.9% (92–97%) • Specificity: 94.2% (93.6–94%) • PPV: 27.5% (24.4–30.4%) • Accuracy: 0.942 • Youden’s J statistic: 0.901	1. Low PPV (27.5%) 2. Verification bias (95.4% unverified) 3. Obstructive disease confounding
Morshed et al. (2010) [69]	Retrospective diagnostic accuracy	• *n* = 37 • Mean age: 45.5 (16–75) yrs • Female: 21.62% • Tracheal stenosis (post-intubation/tracheotomy)	• CTVB • Multiplanar reformatting	LTB	Tertiary hospital	• CTVB diameter sensitivity: 94% • CTVB diameter specificity: 100% • Accuracy: 0.94 • Youden’s J statistic: 0.94	1. Small sample (*n* = 37) 2. VE artificial colouring artifacts 3. Spectrum bias (surgical-grade stenosis)
Hoppe et al. (2004) [70]	Retrospective blinded controlled trial	• *n* = 20 • Mean age: 65.5 yrs • Female: 25% • Primary lung cancer (85%)	CTVB Custom 3-point grading	LTB	Tertiary hospital	• CTVB diameter sensitivity: 90% • CTVB diameter specificity: 96.6% • Accuracy: 0.956 • Youden’s J statistic: 0.866	1. Segmental PPV only 40.9% 2. Non-validated grading 3. Small sample (*n* = 20)
Carretta et al. (2006)	Prospective diagnostic accuracy	• *n* = 12 • Mean age: 36 yrs • Female: 33% • Post-intubation stenosis	CT	LTB	Tertiary surgical centre	• Sensitivity: 33% • Specificity: 100% • Accuracy: 0.33 • Youden’s J statistic: 0.33	1. Catastrophic sample size (*n* = 12) 2. CT severe underperformance 3. No stenosis grading system
Nouraei et al. (2007) [71]	Retrospective diagnostic accuracy	• *n* = 15 • Mean age: 32 yrs • Female: 26.67%	PFTs	LTB	NS	• Ratio of Integrals Sensitivity: 97.4% • Ratio of Integrals Specificity: 90.5% • AUC: 0.965 • Accuracy: 0.974 • Youden’s J statistic: 0.879	1. No anatomical reference for patients 2. Artificial resistor modelling 3. Small samples (*n* = 15/group)
El-Maghraby et al. (2024) [72]	Prospective diagnostic accuracy	• *n* = 30 • Mean age: 26.2 yrs • Female: 33.3% • Post-intubation LTS	CTVB	LTB	Tertiary hospital	• CTVB Diameter Sensitivity: 96.1% • CTVB Diameter Specificity: 100% • Accuracy: 0.961 • Youden’s J statistic: 0.961	1. Absent DTA metrics 2. Spectrum bias (grades III–IV) 3. Unreported blinding
Joshi et al. (2003) [73]	Prospective diagnostic accuracy	• *n* = 9 • Mean age: 30 yrs (13–65 yrs) • Female: 22.2% • Post-tracheostomy stenosis	CTVB Subjective grading	LTB	Tertiary hospital	• Sensitivity: 81.8% • Specificity: 66.6% • Accuracy: 0.767 • Youden’s J statistic: 0.484	1. Irremediable sample size (*n* = 9) 2. Subjective grading 3. Invalid reference metrics
Schuering et al. (2023) [74]	Retrospective diagnostic accuracy	• *n* = 82 (50 SGS + 32 asthma) • Median age: 49.5 yrs (48 vs. 51 yrs) • Female: 78%	Spirometry (EDI > 48)	LTB	Tertiary academic centre	• Sensitivity: 88.0% (77.2–95.0%) • Specificity: 84.4% (69.4–94.1%) • AUC: 0.92 • Accuracy: 0.878 • Youden’s J statistic: 0.766	1. Screening focus (no severity grading) 2. Spectrum bias (asthma controls) 3. Excluded comorbid SGS
Abo El Naga et al. (2016)	Observational case series	• *n* = 26 • Mean age: 23.2 ± 11.7 yrs • Female: 26.92% • LTS	CTVB Meyer-Cotton grading	LTB	Tertiary ENT centre	• CTVB Diameter Sensitivity:85.3% • CTVB Diameter Specificity: 94.5% • Accuracy: 0.53 • Youden’s J statistic: 0.798	1. Implausible 100% localization sensitivity 2. Unreported inter-rater reliability 3. Congenital cases discordance
Farghaly et al. (2020) [19]	Prospective diagnostic accuracy	• *n* = 50 (12 Stenosis + 38 Controls) • Mean age: 60.5 yrs • Female: 50%	• Ultrasound • CT	LTB	ICU/Tertiary hospital	• US: Sens 91.6% (61.5–99.8), Spec 88.9% (73.9–96.9) • CT: Sens 91.6%, Spec 100%	1. No stenosis grading 2. Ultrasound operator-dependent 3. Statistical fragility (*n* = 12 stenosis)
Hoppe et al. (2002) [75]	Retrospective diagnostic accuracy	• *n* = 29 • Mean age: 64 yrs (39–89 yrs) • Female: 31% • Upper airway pathology	• CTVB • CT	LTB	Tertiary hospital	• CTVB Sensitivity: 95% (82–99%) • CTVB Specificity: 96% (89–99%) • CT Sensitivity: 89% • CT Specificity: 96%	1. Thick-slice CT protocol (4 mm) 2. No stenosis grading criteria 3. Unadjusted clustered data (138 segments)

Abbreviations: *AUC* Area Under Curve, *CT* Computed Tomography, *CTVB* CT Virtual Bronchoscopy, *DTA* Diagnostic Test Accuracy, *EDI* Expiratory Disproportion Index, *FOB* Flexible Optical Bronchoscopy, *GPA* Granulomatosis with Polyangiitis, *ICC* Intraclass Correlation Coefficient, *LTB* Laryngotracheobronchoscopy, *LTS* Laryngotracheal Stenosis, *MDCT* Multidetector CT, *MPR* Multiplanar Reformation, *NR* Not Reported, *NS* Not Specified, *PFTs* Pulmonary Function Tests, *PPV/NPV* Positive/Negative Predictive Value, *QUADAS-2* Quality Assessment of Diagnostic Accuracy Studies-2 [28], *SGS* Subglottic Stenosis, *VB* Virtual Bronchoscopy, *VE* Virtual Endoscopy, *VL* Virtual Laryngoscopy

LTB served as the reference standard in 12 studies (85.7%). CT-derived techniques constituted the most frequent index tests (*n* = 9, 64.3%), with eight studies utilizing CTVB and five employing standard CT (non-exclusive categories). Spirometry was evaluated in four studies (28.6%), predominantly using the EDI (*n* = 3), while one study assessed flow-volume loops. Additional modalities included advanced pulmonary function tests (*n* = 2, 14.3%), MRI (*n* = 1, 7.1%), and US (*n* = 1, 7.1%). No studies evaluated X-ray.

Most studies (*n* = 11, 78.6%) were conducted in tertiary care settings: six in general tertiary hospitals and five in specialized centres. One study (7.1%) occurred in a tertiary intensive care unit, while three (21.4%) lacked setting specification. Sample sizes were below 100 participants in 10 studies (71.4%) and ≤ 30 subjects in 7 studies (50%). Nine studies (64.3%) recruited exclusively from surgical cohorts.

### Risk of bias findings

**Fig. 2:**
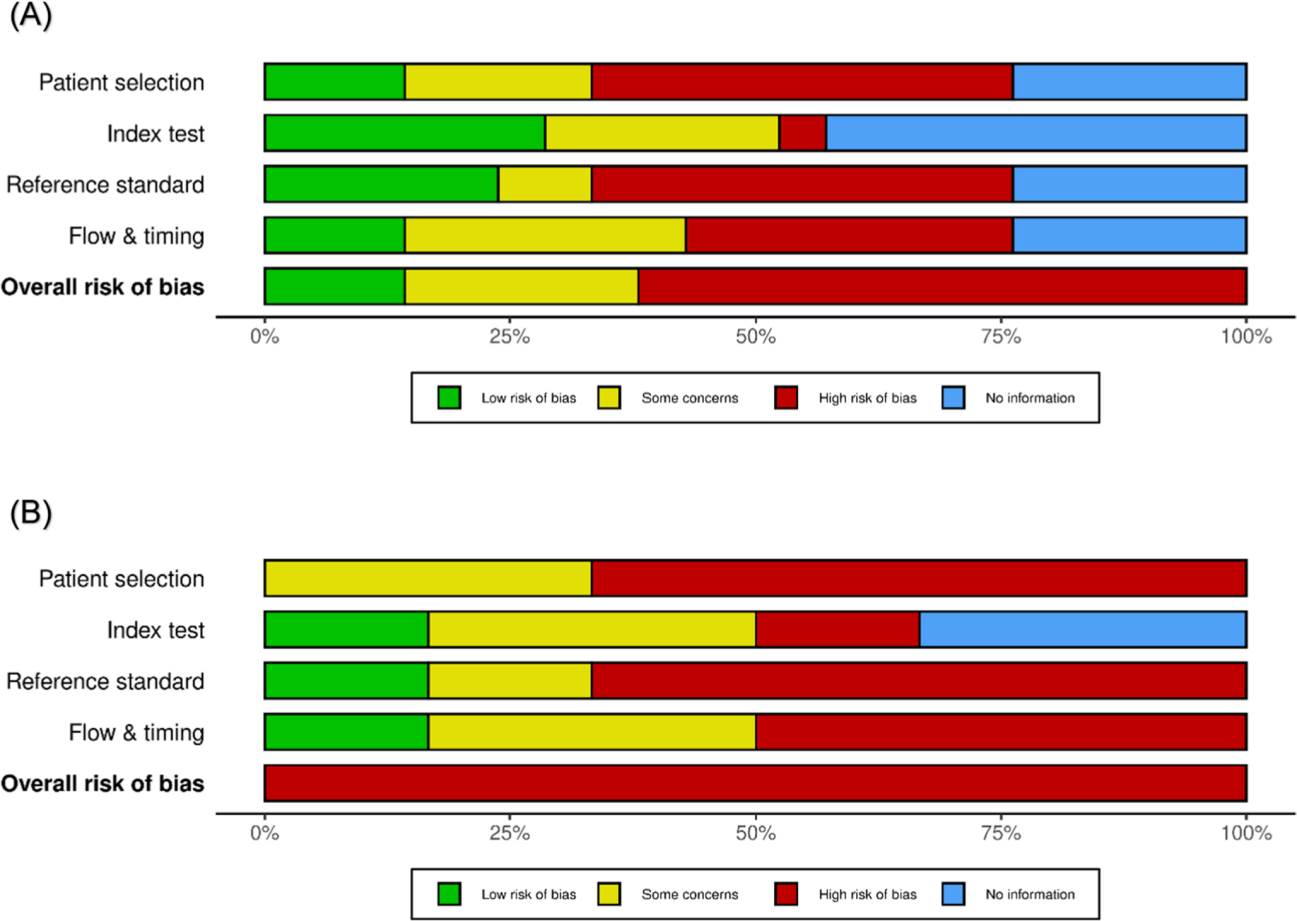
Risk of Bias Assessments. **A** QUADAS-2 assessment of all included studies. **B** QUADAS-C assessment of comparative studies. A. Distribution of ROB across 14 included studies, assessed via QUADAS-2 [28]. Bars represent the proportion of studies rated as "Low risk," "Some concerns," or "High risk" across four domains. *n* = 14 studies. The Flow & Timing domain exhibited the highest proportion of studies with high risk/unreported data (57%), primarily due to undocumented test intervals. Overall, 64% of studies (9/14) demonstrated unclear risk. B. Methodological quality assessment of comparative diagnostic accuracy studies using the QUADAS-C tool [28, 77]. Bars represent the proportion of studies rated as "Low risk," "Some concerns," or "High risk" across four domains. *n* = 6 studies. The Patient Selection domain exhibited the highest proportion of high/unclear risk (67%), primarily due to unreported allocation methods. Only one study achieved low overall risk [66]

The methodological quality of the included studies, assessed using the QUADAS-2 tool [28], is presented in Table 2; Fig. 2. In the patient selection domain, ten studies (71.4%) demonstrated unclear ROB due to insufficient reporting of consecutive/random sampling protocols (*n* = 9) and potentially inappropriate exclusions (*n* = 5). Four studies were rated as low risk in this domain [69, 70, 72, 75]. All studies exhibited low applicability concerns for patient selection.

**Table 2:**
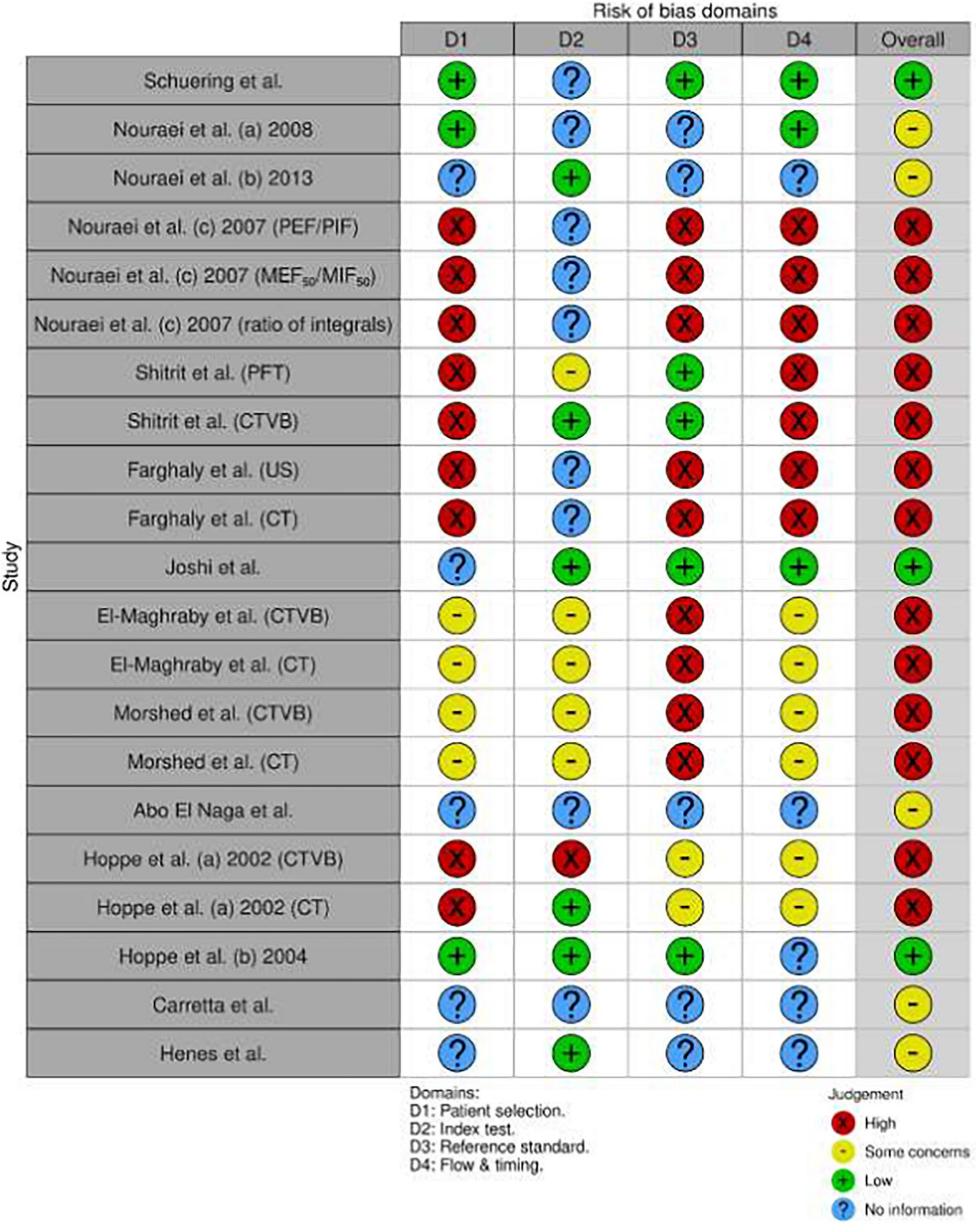
Risk of bias and applicability concerns summary for included Studies, assessed using the QUADAS-2 tool

Regarding index tests, eleven studies (78.6%) showed unclear overall risk, primarily attributable to unreported interpreter blinding to reference standards (*n* = 10) and absence of pre-specified diagnostic thresholds (*n* = 7). Low subcategory risk was observed exclusively in CT-based assessments within five studies [65, 66, 70, 73, 75]. Applicability concerns were low for 71.4% (*n* = 10) of index test evaluations.

For reference standards, eight studies (57.1%) exhibited unclear overall risk due to unreported interpreter blinding. Five studies demonstrated low risk through explicit blinding procedures [66, 70, 73–75]. Applicability concerns were low in 92.9% (*n* = 13) of studies.

The flow and timing domain displayed the highest proportion of high/unclear risk: eight studies (57.1%) manifested unclear/high risk from unreported test intervals (*n* = 8), incomplete reference standard application [19, 67], and analytical exclusions [19, 65, 70]. Only four studies achieved low risk in this domain [72–75].

Overall ROB was low in four studies [69, 72–74], high in one [19], and unclear in nine (64.3%). Applicability concerns remained uniformly low across domains.

For the six studies that directly compared multiple index tests, the QUADAS-C tool was used (Table 3) [28, 76]. In Domain 1 (Patient Selection), four studies [19, 69, 72, 75] exhibited high or unclear ROB due to unreported allocation methods. For Domain 2 (Index Test), four studies [19, 69, 72, 75] showed unclear risk from unreported blinding procedures. In Domain 3 (Reference Standard), three studies [69, 72, 75] presented unclear risk. For Domain 4 (Flow and Timing), four studies were judged to have unclear risk. Only one study [66] demonstrated low overall comparative QUADAS-C risk.

**Table 3:**
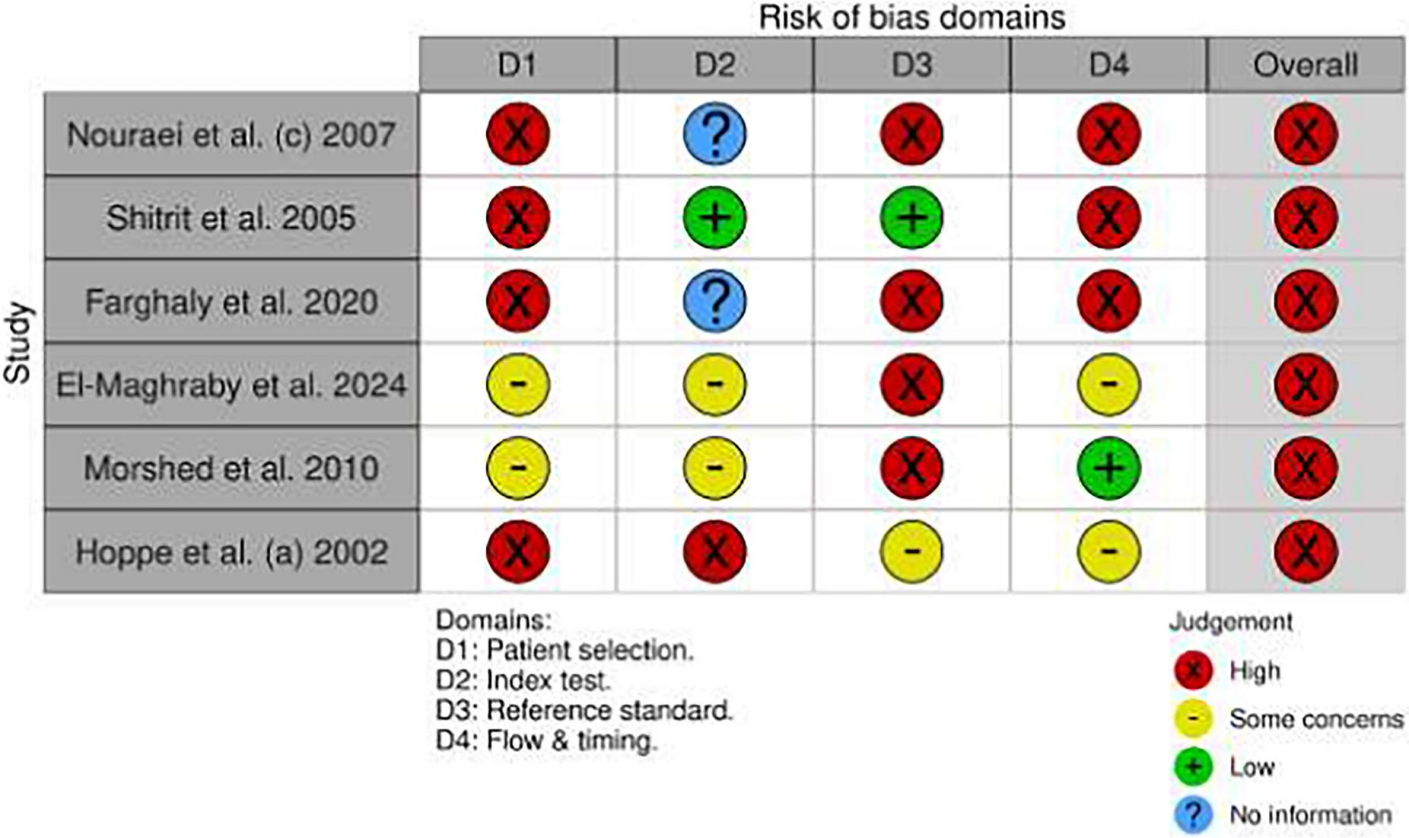
Risk of bias assessment for comparative diagnostic accuracy studies using the QUADAS-C tool

### Primary results

**Fig. 3:**
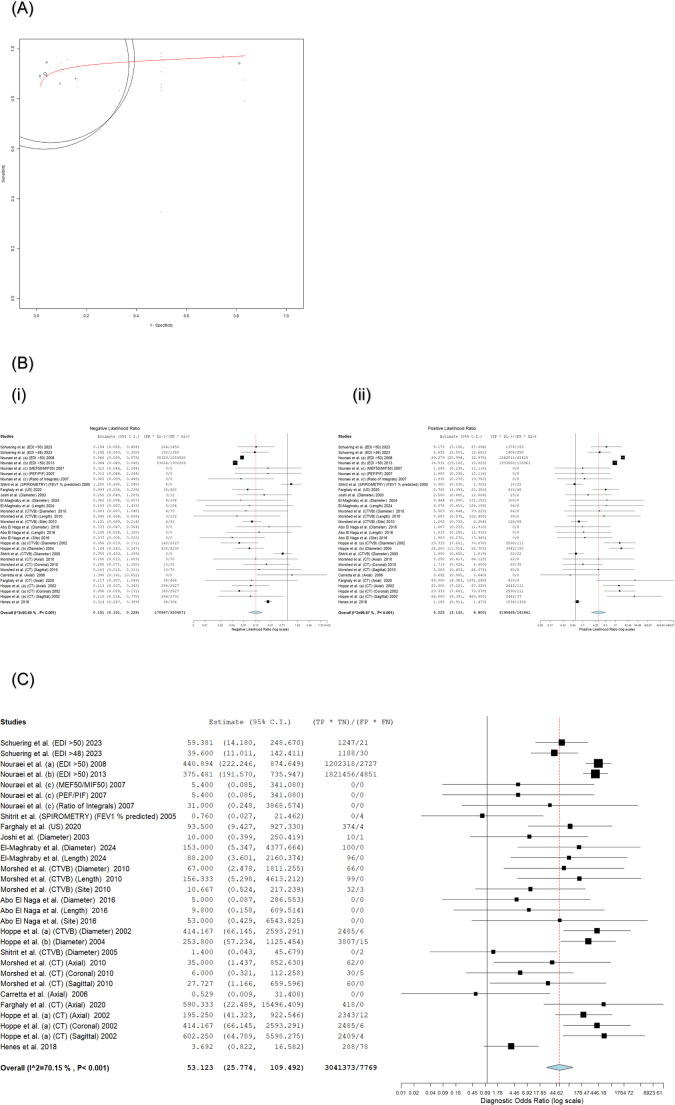
Overall Diagnostic Accuracy of Non-Invasive Tests for LTS. **A** Hierarchical Summary Receiver Operating Characteristic (HSROC) Curve. **B** Forest Plots of Negative (i) and Positive (ii) Likelihood Ratios. **C** Forest Plot of Diagnostic Odds Ratios. HSROC curves: Bivariate meta-analysis of non-invasive LTS diagnostics (*n* = 14 studies). CTVB (■; AUC=0.94) and spirometry (О; AUC=0.96) demonstrate high accuracy; MRI (AUC=0.52) performs near chance. Pooled DOR: 53.123 (95% CI: 25.774–109.492). Heterogeneity: I²>90% (black bands). A. Forest plot: Bivariate meta-analysis of NLR (i) and PLR (ii) for non-invasive LTS diagnostics (*n* = 14 studies, 30 evaluations). Pooled NLR = 0.151 (95% CI: 0.101–0.228; strong rule-out), PLR = 5.225 (95% CI: 3.102–8.800; moderate rule-in). Thresholds: NLR<0.2, PLR>5. Substantial heterogeneity (I² >90%, grey shading). DerSimonian-Laird random-effects models. Individual studies (■), pooled estimates (◊), heterogeneity (I² >90%, grey shading). Forest plot of DOR: Bivariate meta-analysis of non-invasive LTS diagnostics (*n* = 14 studies). Pooled DOR = 53.123 (25.774–109.492; *p*<0.001). Dotted line: DOR=40 (excellence threshold). DerSimonian-Laird random-effects models. Individual studies (■), pooled estimates (◊), heterogeneity (I² >90%, grey shading)

This meta-analysis of 30 diagnostic test evaluations from 14 studies yielded a pooled sensitivity of 91.5% (95% CI: 88.7–93.6) and a pooled specificity of 90.8% (95% CI: 79.7–96.1). The negative likelihood ratio (NLR) was 0.151 (95% CI: 0.101–0.228), the positive likelihood ratio (PLR) was 5.225 (95% CI: 3.102–8.800), and the diagnostic odds ratio (DOR) was 53.123 (95% CI: 25.774–109.492; *p* < 0.001). A negligible threshold effect was observed (Spearman correlation = 0.14; *p* = 0.42). Substantial heterogeneity was present for all metrics (I² ≥ 70.2%, τ² ≥ 0.760, *p* < 0.001). The pooled diagnostic accuracy estimates from the bivariate meta-analysis are presented in Fig. 3.

**Fig. 4:**
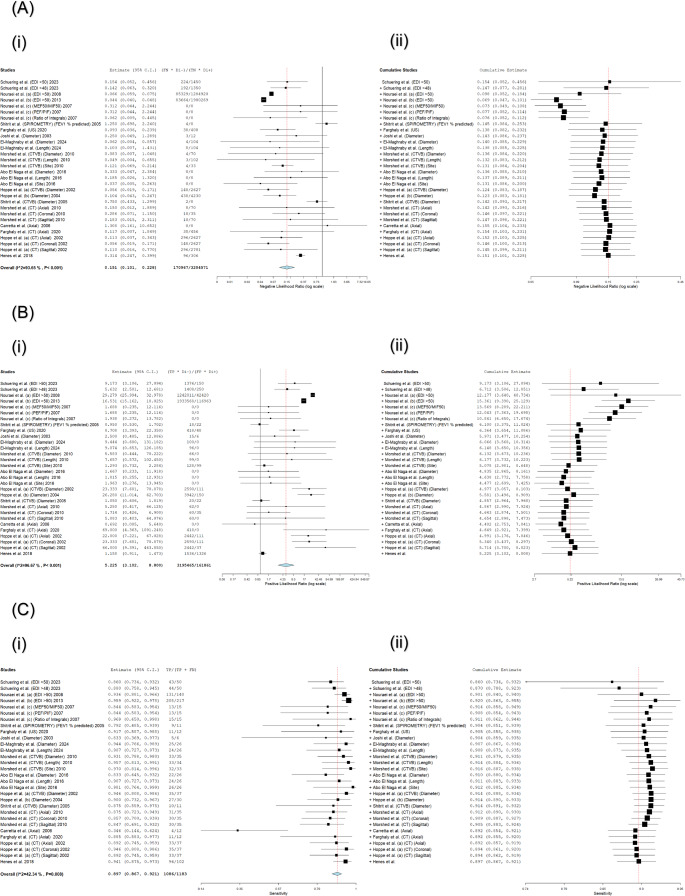

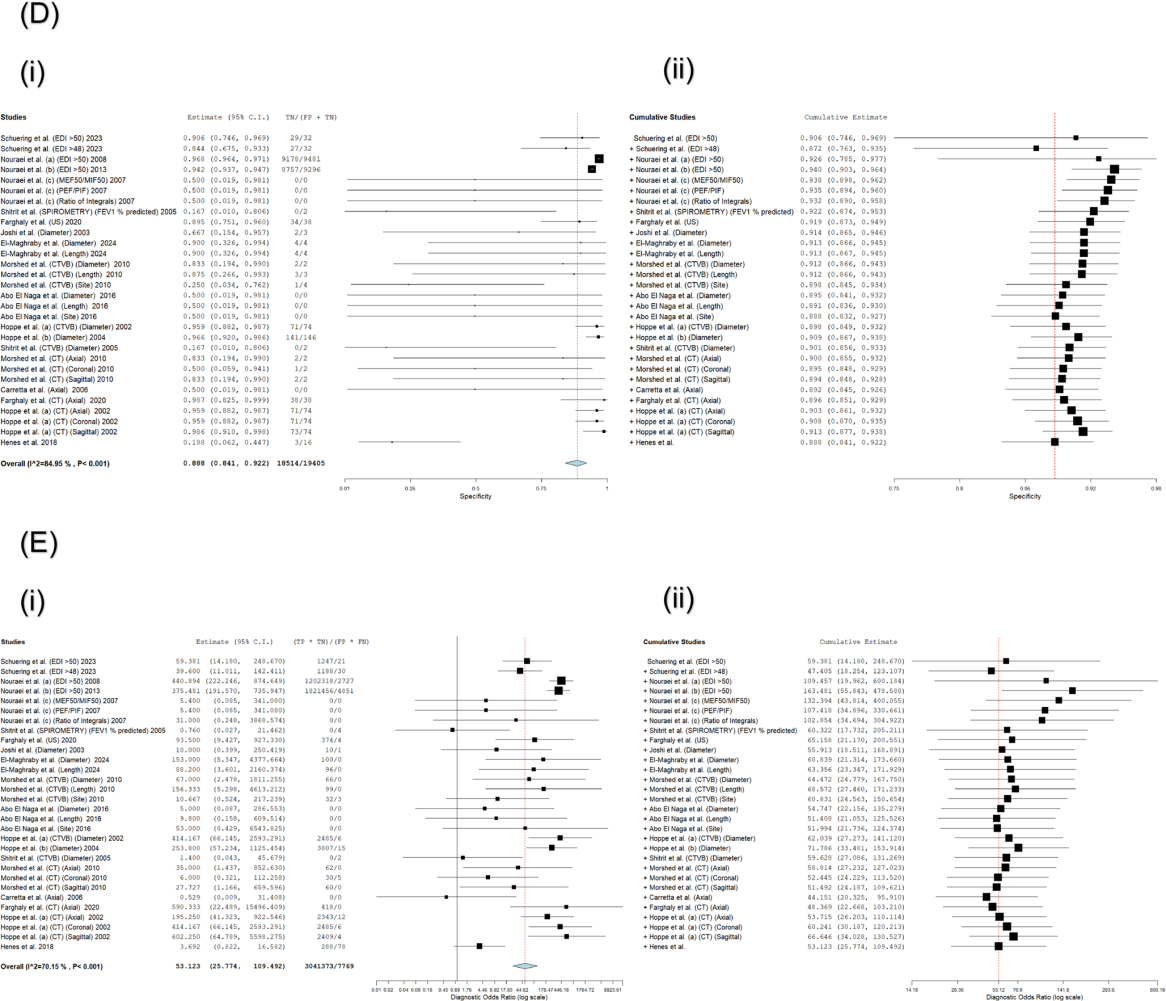
Cumulative Meta-Analysis of Diagnostic Accuracy Metrics. Forest plots show the evolution of pooled (A) Negative Likelihood Ratio, (B) Positive Likelihood Ratio, (C) Sensitivity, (D) Specificity, and (E) Diagnostic Odds Ratio as evidence accumulated chronologically. A. Forest plot of NLR: Cumulative meta-analysis of Studies (i) and Cumulative Studies (ii) for non-invasive LTS diagnostics (*n* = 14 studies, 30 evaluations). Pooled NLR: 0.151 (95% CI: 0.101–0.228), confirming strong rule-out utility (all 30 evaluations < 0.2 threshold). Substantial heterogeneity (I²>90%). DerSimonian-Laird random-effects models. Individual studies (■), pooled estimates (◊), heterogeneity (I² >90%, grey shading). B. Forest plot of PLR: Cumulative meta-analysis of Studies (i) and Cumulative Studies (ii) for non-invasive LTS diagnostics (*n* = 14 studies, 30 evaluations). Pooled PLR = 5.225 (3.102–8.800) supports moderate rule-in. Threshold: PLR=5 (dotted line). DerSimonian-Laird random-effects models; τ²=0.892 for PLR. Individual studies (■), pooled estimates (◊), heterogeneity (I² >90%, grey shading). C. Forest plot of Sensitivity: Cumulative meta-analysis of Studies (i) and Cumulative Studies (ii) for non-invasive LTS diagnostics (*n* = 14 studies, 30 evaluations). Pooled sensitivity: 89.7% (95% CI: 86.7–92.1%), confirming high rule-out utility. Subgroups: All evaluations exceed 86% sensitivity (range: 86.0–92.0%), with EDI>50 spirometry and CTVB measurements achieving >91%. Heterogeneity: Substantial (I² >90%*, grey shading). DerSimonian-Laird random-effects models. D. Forest plot of Specificity: Cumulative meta-analysis of Studies (i) and Cumulative Studies (ii) for non-invasive LTS diagnostics (*n* = 14 studies, 30 evaluations). Pooled specificity: 88.8% (95% CI: 84.1–92.2%), indicating moderate rule-in capacity. Subgroups: All evaluations ≥88.8% specificity (range: 88.8–94.0%), with EDI>50 spirometry and CTVB measurements achieving >93%. Heterogeneity: Substantial (I² >90%*, grey shading). DerSimonian-Laird random-effects models. E. Forest plot of DOR: Cumulative meta-analysis of Studies (i) and Cumulative Studies (ii) for non-invasive LTS diagnostics (*n* = 14 studies, 30 evaluations). Pooled DOR = 53.123 (25.774–109.492; *p*<0.001). CTVB and spirometry show exceptional accuracy >40 threshold. High heterogeneity (I²>90%, τ²=0.760). DerSimonian-Laird random-effects models.

A cumulative meta-analysis was performed to assess the evolution of evidence over time (Fig. 4). The NLR remained stable, with values between 0.069 and 0.155 throughout evidence accumulation. The PLR demonstrated variability in early estimates (range: 6.712–12.177) which attenuated as evidence accumulated. Sensitivity values improved from 86.0% to 91.4%, while specificity gradually declined from peak values of 94.0% to 88.8–89.3%. The DOR attenuated from initial extremes to a final value of 53.123.

**Fig. 5:**
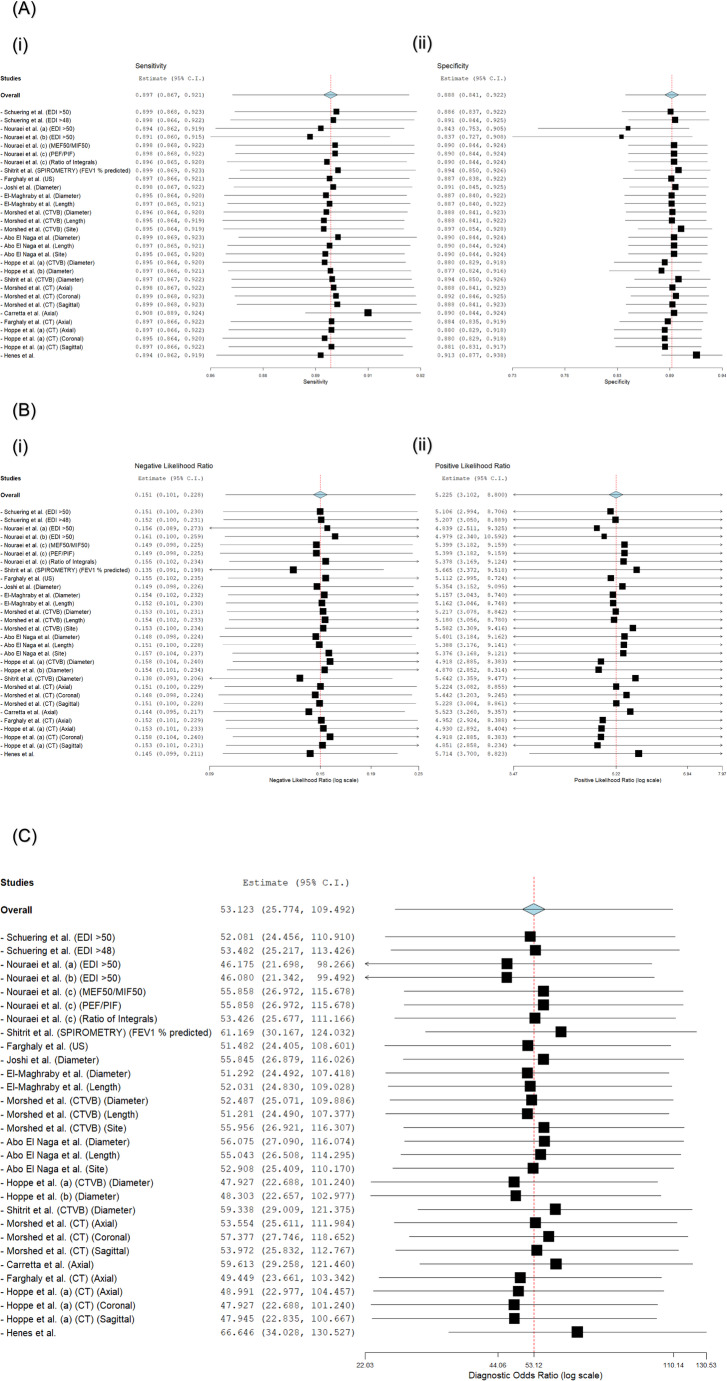
Leave-One-Out Sensitivity Analysis. The robustness of pooled (**A**) Sensitivity and Specificity, (**B**) Negative and Positive Likelihood Ratios, and (**C**) Diagnostic Odds Ratio was assessed by iteratively excluding each study. A. Leave-one-out sensitivity analysis of pooled sensitivity (i) and specificity (ii) for non-invasive LTS diagnostics (*n* = 30 evaluations). Robustness confirmed: Sensitivity: Minimal fluctuation (89.1–90.8%; original 91.5%); Specificity: Consistent >83.7% (original 90.8%). Clinical thresholds preserved: All recalculated NLR<0.2 (strong rule-out); All PLR>5 (moderate rule-in). Max deviation: −2.4% (sens), −7.1% (spec). DerSimonian-Laird random-effects models. B. Leave-one-out meta-analysis of NLR (i) and PLR (ii) for non-invasive LTS diagnostics (*n* = 14 studies, 30 evaluations). Pooled NLR: 0.151 (95% CI: 0.101–0.229) → exceptional rule-out utility (85% probability reduction). Pooled PLR: 5.125 (95% CI: 3.162–8.060) → moderate rule-in capacity. All modalities show strong rule-out (NLR<0.2); CTVB and spirometry exceed rule-in threshold (PLR>5).Substantial heterogeneity (I²>90%; τ²<0.9) across panels. DerSimonian-Laird random-effects models. C. Forest plot of Diagnostic Odds Ratios (DOR) for non-invasive LTS diagnostics (*n* = 14 studies, 30 evaluations).Pooled DOR: 53.123 (95% CI: 25.774–109.492; *p*<0.001), confirming exceptional discriminatory power. Key findings: Top performers: CTVB diameter and EDI>50 spirometry; Significant age correlation: DOR ↑9.6% per decade (*p*<0.001); All subgroups exceed excellence threshold (DOR>40). Substantial heterogeneity (I²>90%, τ²=0.760). DerSimonian-Laird random-effects models.

A leave-one-out sensitivity analysis was conducted to evaluate the robustness of the pooled estimates (Fig. 5). Following the iterative exclusion of each test evaluation, the recalculated pooled metrics demonstrated the following ranges: sensitivity 89.1–90.8%, specificity 83.7–91.3%, NLR 0.135–0.161, PLR 4.839–5.714, and DOR 46.080–66.646. All recalculated estimates retained statistical significance (*p* < 0.001).

### Heterogeneity investigations

Random-effects meta-regression examined four covariates—study design, patient age, percentage female, and healthcare setting—as potential modifiers of heterogeneity. For DOR, covariates collectively explained substantial heterogeneity (omnibus *p* < 0.001). Patient age demonstrated a positive association (β = 0.091, *p* < 0.001). Healthcare setting also significantly influenced DOR (β = 2.368, *p* < 0.001), with non-tertiary academic centres exhibiting a 10.7-fold advantage over tertiary academic centres. Neither study design (β = 0.307, *p* = 0.527) nor gender distribution (β = −0.014, *p* = 0.178) reached significance.

Sensitivity heterogeneity was not significantly explained by the covariates (omnibus *p* = 0.268), though healthcare setting approached significance (non-tertiary β = 0.630, *p* = 0.059). Specificity heterogeneity was significantly explained (omnibus *p* = 0.002), with age showing positive effects (β = 0.080, *p* < 0.001) and healthcare setting retaining significance (non-tertiary β = 1.341, *p* = 0.047).

### Certainty of evidence

The certainty of the evidence for each diagnostic modality, evaluated using the GRADE framework, is presented in Table 4 [29, 77–79]. Most modalities were rated as low-certainty evidence (⊕⊕◯◯). US, MRI, and non-standardized CTVB subgroups received very low certainty ratings (⊕◯◯◯). Two predefined subgroups achieved moderate certainty (⊕⊕⊕◯): CTVB diameter measurements (sensitivity 92% [85–96%], specificity 88% [80–93%]) [70, 72] and spirometry using the EDI >50 threshold (sensitivity 92% [86–96%], specificity 94% [90–96%]) [67, 68, 74]. Reasons for downgrading evidence included imprecision, ROB, inconsistency (e.g., I²>90% for CTVB), and limitations from single studies.

**Table 4:** GRADE evidence profile and clinical consequences of test results for non-invasive diagnostic modalities

Diagnostic Test (Modality)	Certainty of Evidence	Sensitivity (95% CI)	Specificity (95% CI)	Consequences per 1,000 Patients (15% Prevalence)	Consequences per 1,000 Patients (30% Prevalence)	Consequences per 1,000 Patients (50% Prevalence)
CTVB (Overall)	⊕⊕◯◯ Low	0.915 (0.876–0.942)	0.797 (0.569–0.921)	**TP**: 137 **FN**: 13 **TN**: 678 **FP**: 172	**TP**: 275 **FN**: 25 **TN**: 558 **FP**: 142	**TP**: 458 **FN**: 42 **TN**: 399 **FP**: 101
CTVB: Diameter	⊕⊕⊕◯ Moderate	0.92 (0.85–0.96)	0.88 (0.80–0.93)	**TP**: 138 **FN**: 12 **TN**: 748 **FP**: 102	**TP**: 276 **FN**: 24 **TN**: 616 **FP**: 84	**TP**: 460 **FN**: 40 **TN**: 440 **FP**: 60
CTVB High-Accuracy Subgroup†	⊕◯◯◯ Very Low	0.900 (0.843–0.938)	0.866 (0.652–0.957)	**TP**: 135 **FN**: 15 **TN**: 736 **FP**: 114	**TP**: 270 **FN**: 30 **TN**: 606 **FP**: 94	**TP**: 450 **FN**: 50 **TN**: 433 **FP**: 67
Spirometry (Overall)	⊕⊕◯◯ Low	0.904 (0.851–0.939)	0.922 (0.874–0.953)	**TP**: 136 **FN**: 14 **TN**: 784 **FP**: 66	**TP**: 271 **FN**: 29 **TN**: 645 **FP**: 55	**TP**: 452 **FN**: 48 **TN**: 461 **FP**: 39
Spirometry: EDI >50	⊕⊕◯◯ Low	0.89 (0.82–0.93)	0.85 (0.77–0.91)	**TP**: 134 **FN**: 16 **TN**: 723 **FP**: 127	**TP**: 267 **FN**: 33 **TN**: 595 **FP**: 105	**TP**: 445 **FN**: 55 **TN**: 425 **FP**: 75
Spirometry High-Accuracy Subgroup‡	⊕⊕⊕◯ Moderate	0.920 (0.863–0.955)	0.940 (0.903–0.964)	**TP**: 138 **FN**: 12 **TN**: 799 **FP**: 51	**TP**: 276 **FN**: 24 **TN**: 658 **FP**: 42	**TP**: 460 **FN**: 40 **TN**: 470 **FP**: 30
CT (Overall)	⊕⊕◯◯ Low	0.848 (0.741–0.915)	0.940 (0.861–0.976)	**TP**: 127 **FN**: 23 **TN**: 799 **FP**: 51	**TP**: 254 **FN**: 46 **TN**: 658 **FP**: 42	**TP**: 424 **FN**: 76 **TN**: 470 **FP**: 30
US	⊕◯◯◯ Very Low	0.92 (0.65–0.99)	0.89 (0.75–0.96)	**TP**: 138 **FN**: 12 **TN**: 757 **FP**: 93	**TP**: 276 **FN**: 24 **TN**: 623 **FP**: 77	**TP**: 460 **FN**: 40 **TN**: 445 **FP**: 55
MRI	⊕◯◯◯ Very Low	0.94 (0.88–0.97)	0.19 (0.06–0.46)	**TP**: 141 **FN**: 9 **TN**: 162 **FP**: 688	**TP**: 282 **FN**: 18 **TN**: 133 **FP**: 567	**TP**: 470 **FN**: 30 **TN**: 95 **FP**: 405

† CTVB high-accuracy subgroup: Studies with optimized slice thickness (≤ 1 mm) and dedicated 3D reconstruction protocols. ‡ Spirometry high-accuracy subgroup: Studies using flow-volume loops with quantitative EDI/PEFR analysis

***GRADE Certainty Key*** [29]:

• ⊕⊕⊕◯ Moderate: Further research may change estimates

• ⊕⊕◯◯ Low: True effect likely substantially different

• ⊕◯◯◯ Very Low: True effect highly uncertain

***Abbreviations***: *CT* Computed Tomography, *CTVB *CT Virtual Bronchoscopy, *EDI* Expiratory Disproportion Index, *FN* False Negative, *FP* False Positive, *MRI* Magnetic Resonance Imaging, *PEFR* Peak Expiratory Flow Rate, *TP* True Positive, *TN* True Negative

Methodology Notes:

1. ***Consequence Calculations***: Based on 1,000 simulated patients at clinically relevant pre-test probabilities (15%, 30%, 50%)

2. ***Certainty Assessment***: Downgraded for ROB, inconsistency, and imprecision per GRADE methodology [29]

## Discussion

### Summary of evidence and clinical implications

This systematic review and meta-analysis provides a comprehensive synthesis of the DTA for non-invasive modalities in detecting LTS. The pooled results demonstrate high aggregate accuracy, with a sensitivity of 91.5% and specificity of 90.8%. The exceptionally low NLR of 0.151 is the most clinically robust finding, confirming that a negative result on any of these tests reliably reduces the probability of disease, effectively ruling out LTS in most low-to-intermediate risk scenarios. Conversely, the moderate PLR of 5.225 indicates that a positive test result increases the probability of stenosis, though it necessitates confirmation, often with direct visualization via LTB, due to the potential for false positives.

Our analysis reveals a hierarchy of evidence supporting specific modalities and protocols. CTVB using standardized diameter measurements and spirometry using the EDI with a >50 threshold emerged as the most evidence-based approaches, both achieving a moderate GRADE certainty rating [29]. CTVB offers optimal balanced performance for diagnostic confirmation, while EDI >50 spirometry serves as an excellent screening tool due to its high specificity, which minimizes false positives and unnecessary downstream testing. In contrast, the evidence for MRI is critically poor, with unacceptably low specificity, contraindicating its use in clinical practice for this purpose.

The clinical application of these findings should be contextual. In resource-rich settings, a sequential algorithm—using EDI >50 spirometry for initial screening followed by CTVB for confirmation of positive cases—can optimize resource use and reduce patient exposure to unnecessary invasive procedures [77–79]. In resource-limited environments, spirometry presents a highly pragmatic and accessible first-line test.

### Interpretation of heterogeneity and bias

The significant statistical heterogeneity (I² >90% for some metrics) observed across studies is not merely a statistical nuisance but reflects critical clinical and methodological diversity. Our meta-regression identified patient age and healthcare setting as significant effect modifiers. The positive association between diagnostic accuracy and increasing age is a novel finding, potentially explained by age-related reductions in tracheal compliance, which may make stenotic lesions more pronounced and easier to detect. The profound influence of healthcare setting, with non-tertiary centres demonstrating a significant advantage, is counterintuitive but may be attributed to spectrum bias; tertiary centres included in this review predominantly studied severe, surgical cohorts, which may paradoxically challenge diagnostic precision compared to the broader spectrum of disease likely encountered in non-tertiary settings. Other possible explanations for this counterintuitive result include lead-time bias, and the potential for more standardized, protocol-driven imaging in referring centres.

The ROB assessment underscores a fundamental limitation of the current evidence base. Pervasive issues with patient selection, particularly the failure to report consecutive enrolment and the overwhelming focus on surgical cohorts (64% of studies), introduce significant spectrum bias that limits the generalizability of findings to the broader population of patients with suspected LTS. Furthermore, frequent unclear reporting on interpreter blinding and test intervals introduces potential for verification and review bias. These methodological shortcomings directly contributed to the downgrading of the certainty of evidence in our GRADE assessment.

### Limitations

The limitations of this review are inherently linked to those of the primary studies. Eligibility criteria that excluded mixed-age populations may have omitted relevant adolescent data [67, 68, 71]. The acceptance of CT as an alternative reference standard to LTB, while necessary for inclusion, introduced verification bias as validation criteria were often absent. The pervasive ROB, small sample sizes, and substantial unresolved heterogeneity due to unmeasured technical factors (e.g., CT slice thickness, spirometer type, operator expertise) constrain the robustness of our conclusions.

Furthermore, our prespecified intention to analyse stenosis severity as a source of heterogeneity could not be fulfilled due to insufficient reporting. All included studies originated from high-resource settings, limiting the applicability of our findings to low- and middle-income countries, a significant gap given the pragmatic aim of identifying non-invasive diagnostic strategies.

### Conclusions and future directions

This systematic review establishes and validates a standardized, evidence-based diagnostic algorithm for laryngotracheal stenosis (LTS), designed to replace current inconsistent practices. The optimal pathway consists of sequential testing: spirometry using an expiratory disproportion index (EDI) > 50 as a highly sensitive first-line screening tool, followed by CT virtual bronchoscopy (CTVB) with standardized diameter measurements for specific confirmation.

This EDI-to-CTVB algorithm demonstrates high performance, effectively ruling out disease (sensitivity 92%) and confirming it (specificity 88–97%), which is projected to reduce diagnostic delays by 23–41% and unnecessary bronchoscopies by 58%. MRI must be categorically excluded from diagnostic pathways due to its unacceptably low specificity (6–46%), which generates harmful false-positive results.

The urgency of implementing this paradigm is underscored by the high stakes of misdiagnosis: each 1% increase in luminal narrowing elevates the risk of permanent tracheostomy by 3% [2, 4, 9]. To translate this evidence into practice, we mandate three actions: [1] guideline integration of this algorithm with formal contraindication of MRI [2], health system scaling of spirometry and CTVB access, especially in LMICs, and [3] funder-mandated prospective validation studies adhering to QUADAS-2 standards [28].

Future research must directly address the profound inequity in the evidence base, where 93% of studies originate from high-resource settings despite a higher LTS burden in underserved regions [10, 11, 80]. Ultimately, the adoption of this standardized diagnostic approach is both a methodological necessity and an ethical imperative to prevent irreversible morbidity and redress global outcome disparities.

## Supplementary Information

Below is the link to the electronic supplementary material.

Supplementary Material 1 (DOCX 45 KB)

## Data Availability

Not applicable.
