# Supplementary material for: Diagnostic accuracy of non-invasive modalities for laryngotracheal stenosis: A systematic review and meta-Analysis
Source: Eur Arch Otorhinolaryngol. 2025 Oct 24;283(3):1423–41. doi: 10.1007/s00405-025-09773-3 (PMC13002695; doi:10.1007/s00405-025-09773-3)
Supplement: Supplementary file 1 — Supplementary Material 1 (DOCX 45 KB) [file 405_2025_9773_MOESM1_ESM.docx]

***Supplementary Material 1:*** *MEDLINE/PubMed Search Strategy.*

| ***Draft Search Strategy for MEDLINE (via PubMed)*** |
| --- |
| ***1. Population (Adolescent, Adult, Laryngotracheal Stenosis, Related Conditions)*** |
| (Adolescent[MeSH] OR Adult[MeSH])  AND  (Laryngostenosis[MeSH] OR Tracheal Stenosis[MeSH] OR Airway Obstruction[MeSH] OR Tracheomalacia[MeSH] OR Laryngeal Diseases[MeSH] OR Constriction, Pathologic[MeSH] OR Tracheal Diseases[MeSH] OR Respiratory Disorders[MeSH]) |
| ***2. Index Tests (Diagnostic Techniques and Imaging)*** |
| (Diagnostic Techniques and Procedures[MeSH] OR Diagnostic Tests, Routine[MeSH] OR X-Rays[MeSH] OR Respiratory Function Tests[MeSH] OR Spirometry[MeSH] OR Ultrasonography[MeSH] OR Imaging[MeSH] OR Tomography, X-Ray Computed[MeSH] OR Tomography[MeSH] OR Imaging, Three-Dimensional[MeSH] OR Magnetic Resonance Imaging[MeSH]) |
| ***3. Comparator Tests (Reference Standards)*** |
| (Reference Standards[MeSH] OR Laryngoscopy[MeSH] OR Bronchoscopy[MeSH] OR Endoscopy[MeSH]) |
| ***4. Outcomes / Study Designs (Diagnostic Accuracy Study Types)*** |
| (Retrospective Studies[MeSH] OR Prospective Studies[MeSH] OR Cross-Sectional Studies[MeSH] OR Randomized Controlled Trials as Topic[MeSH] OR Triage[MeSH]) |
| ***5. Combine All Components (PubMed/MEDLINE Syntax)*** |
| #Population AND #Index Tests AND #Comparator Tests AND #Outcomes:  (Adolescent[MeSH]*[tiab] OR Adult[MeSH]*[tiab])  AND  (Laryngostenosis[MeSH]*[tiab] OR Tracheal Stenosis[MeSH]*[tiab] OR Airway Obstruction[MeSH]*[tiab] OR Tracheomalacia[MeSH]*[tiab] OR Laryngeal Diseases[MeSH]*[tiab] OR Constriction, Pathologic[MeSH]*[tiab] OR Tracheal Diseases[MeSH]*[tiab] OR Respiratory Disorders[MeSH]*[tiab])  AND  (Diagnostic Techniques and Procedures[MeSH]*[tiab] OR Diagnostic Tests, Routine[MeSH]*[tiab] OR X-Rays[MeSH]*[tiab] OR Respiratory Function Tests[MeSH]*[tiab] OR Spirometry[MeSH]*[tiab] OR Ultrasonography[MeSH]*[tiab] OR Imaging[MeSH]*[tiab] OR Tomography, X-Ray Computed[MeSH]*[tiab] OR Tomography[MeSH]*[tiab]OR Imaging, Three-Dimensional[MeSH]*[tiab] OR Magnetic Resonance Imaging[MeSH]*[tiab])  AND  (Reference Standards[MeSH]*[tiab] OR Laryngoscopy[MeSH]*[tiab] OR Bronchoscopy[MeSH]*[tiab] OR Endoscopy[MeSH]*[tiab])  AND  (Retrospective Studies[MeSH]*[tiab] OR Prospective Studies[MeSH]*[tiab] OR Cross-Sectional Studies[MeSH]*[tiab] OR Randomized Controlled Trials as Topic[MeSH]*[tiab] OR Triage[MeSH]*[tiab]) |
| ***Draft Search Strategy for Embase*** |
| ***1. Population (Adolescent, Adult, Laryngotracheal Stenosis, Related Conditions)*** |
| (adolescent/ OR adult/) AND (laryngotracheal stenosis/ OR laryngeal stenosis/ OR tracheal stenosis/ OR subglottic stenosis/ OR glottic stenosis/ OR airway obstruction/ OR respiratory tract obstruction/ OR tracheomalacia/) |
| ***2. Index Tests (Diagnostic Techniques and Imaging)*** |
| (diagnostic imaging/ OR x-ray/ OR radiography/ OR spirometry/ OR pulmonary function test/ OR ultrasound/ OR computed tomography/ OR three-dimensional imaging/ OR magnetic resonance imaging/) |
| ***3. Comparator Tests (Reference Standards)*** |
| (laryngoscopy/ OR bronchoscopy/ OR endoscopy/) |
| ***4. Outcomes / Study Designs (Diagnostic Accuracy Study Types)*** |
| (retrospective study/ OR prospective study/ OR cross-sectional study/ OR randomized controlled trial/ OR diagnostic accuracy/ OR likelihood function/ OR triage/) |
| ***5. Combine All Components (Embase Syntax)*** |
| #Population AND #Index Tests AND #Comparator Tests AND #Outcomes:  ((adolescent/ OR adult/)  AND  (laryngotracheal stenosis/ OR laryngeal stenosis/ OR tracheal stenosis/ OR subglottic stenosis/ OR glottic stenosis/ OR airway obstruction/ OR respiratory tract obstruction/ OR tracheomalacia/))  AND  (diagnostic imaging/ OR x-ray/ OR radiography/ OR spirometry/ OR pulmonary function test/ OR ultrasound/ OR computed tomography/ OR three-dimensional imaging/ OR magnetic resonance imaging/)  AND  (laryngoscopy/ OR bronchoscopy/ OR endoscopy/)  AND  (retrospective study/ OR prospective study/ OR cross-sectional study/ OR randomized controlled trial/ OR diagnostic accuracy/ OR likelihood function/ OR triage/) |

**Supplementary Material 2:** Embase Search Strategy.

| ***Draft Search Strategy for Embase*** |
| --- |
| ***1. Population (Adolescent, Adult, Laryngotracheal Stenosis, Related Conditions)*** |
| (adolescent/ OR adult/) AND (laryngotracheal stenosis/ OR laryngeal stenosis/ OR tracheal stenosis/ OR subglottic stenosis/ OR glottic stenosis/ OR airway obstruction/ OR respiratory tract obstruction/ OR tracheomalacia/) |
| ***2. Index Tests (Diagnostic Techniques and Imaging)*** |
| (diagnostic imaging/ OR x-ray/ OR radiography/ OR spirometry/ OR pulmonary function test/ OR ultrasound/ OR computed tomography/ OR three-dimensional imaging/ OR magnetic resonance imaging/) |
| ***3. Comparator Tests (Reference Standards)*** |
| (laryngoscopy/ OR bronchoscopy/ OR endoscopy/) |
| ***4. Outcomes / Study Designs (Diagnostic Accuracy Study Types)*** |
| (retrospective study/ OR prospective study/ OR cross-sectional study/ OR randomized controlled trial/ OR diagnostic accuracy/ OR likelihood function/ OR triage/) |
| ***5. Combine All Components (Embase Syntax)*** |
| #Population AND #Index Tests AND #Comparator Tests AND #Outcomes:  ((adolescent/ OR adult/)  AND  (laryngotracheal stenosis/ OR laryngeal stenosis/ OR tracheal stenosis/ OR subglottic stenosis/ OR glottic stenosis/ OR airway obstruction/ OR respiratory tract obstruction/ OR tracheomalacia/))  AND  (diagnostic imaging/ OR x-ray/ OR radiography/ OR spirometry/ OR pulmonary function test/ OR ultrasound/ OR computed tomography/ OR three-dimensional imaging/ OR magnetic resonance imaging/)  AND  (laryngoscopy/ OR bronchoscopy/ OR endoscopy/)  AND  (retrospective study/ OR prospective study/ OR cross-sectional study/ OR randomized controlled trial/ OR diagnostic accuracy/ OR likelihood function/ OR triage/) |

**Supplementary Material 3:** CINAHL (EBSCOhost) Search Strategy.

| ***Draft Search Strategy for CINAHL*** |
| --- |
| ***1. Population (Laryngotracheal Stenosis and Related Conditions)*** |
| (MH "Laryngotracheal Stenosis") OR  (MH "Laryngeal Diseases") OR  (tracheal diseases) OR  (MH "Airway Obstruction") OR  (MH "Respiratory Tract Diseases") |
| ***2. Index Tests (Diagnostic Tests and Imaging)*** |
| (MH "Diagnostic Tests, Routine") OR  (MH "Diagnostic Imaging") OR  (MH "Radiography") OR  (MH "Spirometry") OR  (MH "Respiratory Function Tests") OR  (MH "Ultrasonography") OR  (MH "Tomography, X-Ray Computed") OR  (MH "Imaging, Three-Dimensional") OR  (MH "Magnetic Resonance Imaging") |
| ***3. Comparator Tests (Reference Standards)*** |
| (MH "Laryngoscopy") OR  (MH "Bronchoscopy") OR  (MH "Endoscopy") OR  (MH "Reference Standards") |
| ***4. Outcomes / Study Designs*** |
| (MH "Retrospective Studies") OR  (MH "Prospective Studies") OR  (MH "Cross-Sectional Studies") OR  (MH "Randomized Controlled Trials") OR  (MH "Triage") |
| ***5. Combine Concepts (EBSCOhost Syntax)*** |
| (Population) AND (Index Tests) AND (Comparator Tests) AND (Outcomes):  1. (MH "Laryngotracheal Stenosis") OR (MH "Laryngeal Diseases") OR (tracheal diseases).ti,ab. OR (MH "Airway Obstruction") OR (MH "Respiratory Tract Diseases")  2. (MH "Diagnostic Tests, Routine") OR (MH "Diagnostic Imaging") OR (MH "Radiography") OR (MH "Spirometry") OR (MH "Respiratory Function Tests") OR (MH "Ultrasonography") OR (MH "Tomography, X-Ray Computed") OR (MH "Imaging, Three-Dimensional") OR (MH "Magnetic Resonance Imaging")  3. (MH "Laryngoscopy") OR (MH "Bronchoscopy") OR (MH "Endoscopy") OR (MH "Reference Standards")  4. (MH "Retrospective Studies") OR (MH "Prospective Studies") OR (MH "Cross-Sectional Studies") OR (MH "Randomized Controlled Trials") OR (MH "Triage")  5. 1 AND 2 AND 3 AND 4 |

**Supplementary Material 4:** Free-Text Keyword Strategy.

| ***Draft Search Strategy for FREE TEXT KEYWORDS*** |
| --- |
| ***1. Population (Laryngotracheal Stenosis and Related Conditions)*** |
| "adolescent*" OR "teen*" OR "youth*" OR "young adult*" OR "adult*" OR "mature patient*" OR "older child*" OR "post-pubertal*" OR "postpubertal*"  "laryngotracheal stenosis" OR "laryngotracheal" OR "laryngostenosis" OR "tracheal stenosis" OR "subglottic stenosis" OR "glottic stenosis" OR "supraglottic stenosis" OR "airway stenosis" OR "airway narrowing" OR "airway obstruction" OR "central airway stenosis" OR "respiratory tract obstruction" OR "upper airway obstruction" OR "tracheomalacia"  "LTS" OR "AALTS" |
| ***2. Index Tests (Diagnostic Tests and Imaging)*** |
| "x-ray*" OR "radiograph*" OR "plain film*" OR "chest x-ray*" OR "neck x-ray*"  "spirometry" OR "spirometric" OR "pulmonary function test*" OR "PFT*" OR "flow-volume loop*" OR "peak expiratory flow"  "ultrasound" OR "ultrasonography" OR "sonography" OR "sonographic"  "computed tomography" OR "CT scan*" OR "CT imaging" OR "dynamic expiratory CT" OR "multidetector CT" OR "MDCT"  "virtual bronchoscopy" OR "VB"  "magnetic resonance imaging" OR "MRI" OR "MR imaging"  "diagnostic imaging" OR "imaging method*" OR "3D imaging" OR "three dimensional imaging" |
| ***3. Comparator Tests (Reference Standards)*** |
| "laryngotracheobronchoscopy" OR "laryngoscopy" OR "bronchoscopy" OR "endoscopy"  "flexible laryngoscopy" OR "flexible bronchoscopy" OR "direct laryngoscopy" OR "microlaryngoscopy" OR "videolaryngoscopy"  "reference standard" OR "gold standard" |
| ***4. Outcomes / Study Designs*** |
| "diagnostic accuracy" OR "diagnostic performance" OR "sensitivity" OR "specificity" OR "positive predictive value" OR "negative predictive value" OR "likelihood ratio" OR "ROC curve" OR "receiver operating characteristic" OR "area under the curve" OR "AUC" OR "false positive*" OR "false negative*" OR "true positive*" OR "true negative*" |
| ***5. Study Design/Methodology Terms*** |
| "systematic review" OR "meta-analysis" OR "cross-sectional" OR "cohort" OR "prospective" OR "retrospective" OR "randomized controlled trial" OR "RCT" OR "diagnostic test accuracy" OR "DTA" OR "comparative study" OR "QUADAS" OR "GRADE" |

**Supplementary Material 5:** Characteristics and Exclusion Rationales for Studies Excluded at Full-Text Review.

| **Study Author(s) (Year)** | **Primary Exclusion Reason(s)** | **Secondary Exclusion Reason(s)** |
| --- | --- | --- |
| Taha et al. (2009) [81] | - Insufficient age verification (≥12 years) or inclusion of paediatric cohorts | - Inadequate documentation of prior surgical intervention for LTS |
| Nikolova (2018) [82] | - Utilization of FOB instead of prespecified standards (LTB/CT) - Absence of reported sensitivity/specificity metrics or sufficient data for derivation | - Assessment beyond laryngotracheal boundaries or insufficient regional specification - Inadequate documentation of prior surgical intervention for LTS |
| Koletsis et al. (2007) [83] | - Insufficient age verification (≥12 years) or inclusion of paediatric cohorts - Utilization of FOB instead of prespecified standards (LTB/CT) - Absence of reported sensitivity/specificity metrics or sufficient data for derivation | - Inadequate documentation of prior surgical intervention for LTS - Assessment beyond laryngotracheal boundaries or insufficient regional specification |
| Parshin et al. (2016) [84] | - Absence of reported sensitivity/specificity metrics or sufficient data for derivation - Insufficient age verification (≥12 years) or inclusion of paediatric cohorts | - Inadequate documentation of prior surgical intervention for LTS |
| Thenappan et al. (2023) [85] | - Absence of reported sensitivity/specificity metrics or sufficient data for derivation - Utilization of FOB instead of prespecified standards (LTB/CT) - Insufficient age verification (≥12 years) or inclusion of paediatric cohorts | - Inadequate documentation of prior surgical intervention for LTS |
| Song et al. (2006) [86] | - Insufficient age verification (≥12 years) or inclusion of paediatric cohorts - Assessment beyond laryngotracheal boundaries or insufficient regional specification | - Inadequate documentation of prior surgical intervention for LTS |
| Sundarakumar et al. (2011) [87] | - Utilization of FOB instead of prespecified standards (LTB/CT) - Insufficient age verification (≥12 years) or inclusion of paediatric cohorts - Absence of reported sensitivity/specificity metrics or sufficient data for derivation | - Assessment beyond laryngotracheal boundaries or insufficient regional specification |
| Hall et al. (2017) [88] | - Utilization of FOB instead of prespecified standards (LTB/CT) - Absence of reported sensitivity/specificity metrics or sufficient data for derivation - Insufficient age verification (≥12 years) or inclusion of paediatric cohorts | - Emphasis on technical validation/therapeutic outcomes or methodological ambiguity - Inadequate documentation of prior surgical intervention for LTS |
| Sorantin et al. (2003) [92] | - Emphasis on technical validation/therapeutic outcomes or methodological ambiguity - Absence of reported sensitivity/specificity metrics or sufficient data for derivation | - Insufficient age verification (≥12 years) or inclusion of paediatric cohorts - Inadequate documentation of prior surgical intervention for LTS |
| Heyer et al. (2007) | - Insufficient age verification (≥12 years) or inclusion of paediatric cohorts - Assessment beyond laryngotracheal boundaries or insufficient regional specification - Utilization of FOB instead of prespecified standards (LTB/CT) | - Inadequate documentation of prior surgical intervention for LTS |
| Luccichenti et al. (2003) [89] | - Absence of reported sensitivity/specificity metrics or sufficient data for derivation - Indeterminate LTS diagnostic focus - Emphasis on technical validation/therapeutic outcomes or methodological ambiguity | - Assessment beyond laryngotracheal boundaries or insufficient regional specification - Inadequate documentation of prior surgical intervention for LTS |
| García-Buenrostro & González-Palafox (2005) [90] | - Insufficient age verification (≥12 years) or inclusion of paediatric cohorts - Absence of reported sensitivity/specificity metrics or sufficient data for derivation | - Not applicable |
| Lahjaouj et al. 2021 [91] | - Emphasis on technical validation/therapeutic outcomes or methodological ambiguity - Absence of reported sensitivity/specificity metrics or sufficient data for derivation | - Insufficient age verification (≥12 years) or inclusion of paediatric cohorts - Inadequate documentation of prior surgical intervention for LTS |

Studies listed advanced beyond title/abstract screening but violated ≥1 eligibility criterion during full-text review. Primary rationales represent definitive exclusion triggers; secondary indicate additional methodological concerns. Abbreviations: FOB = Fibreoptic Bronchoscopy; LTB = Laryngotracheobronchoscopy (46,82–93)

**REFERENCES**

81. Taha MS, Mostafa BE, Fahmy M, Ghaffar MKA, Ghany EA. Spiral CT virtual bronchoscopy with multiplanar reformatting in the evaluation of post-intubation tracheal stenosis: comparison between endoscopic, radiological and surgical findings. European Archives of Oto-Rhino-Laryngology. 2009 Jun 12;266(6):863–6.

82. Nikolova S. Advanced and Non- Invasive Technologies for Imaging and Visualization of Obstructive Lesions of the Tracheobronchial Tree- Virtual Bronchoscopy. Vol. 23, KNOWLEDGE-International Journal. 2018.

83. Koletsis EN, Kalogeropoulou C, Prodromaki E, Kagadis GC, Katsanos K, Spiropoulos K, et al. Tumoral and non-tumoral trachea stenoses: evaluation with three-dimensional CT and virtual bronchoscopy. J Cardiothorac Surg. 2007 Dec 12;2(1):18.

84. Parshin VD, Koroleva IM, Mishchenko MA. Evolution of diagnostic methods for cicatrical tracheal stenosis and tracheomalacia. Khirurgiya Zhurnal im NI Pirogova. 2016;(5):17.

85. Thenappan T, Vivekanandan B, Somu L. Study to establish fiberoptic laryngoscopy as a diagnostic and investigation tool for laryngotracheal stenosis and to use the same for prognosis of the disease: retrospective analysis. International Journal of Advances in Medicine. 2023 Jul 26;10(8):607–10.

86. Song SA, Franco RA. Serial intralesional steroid injection for subglottic stenosis. Laryngoscope. 2020 Mar 20;130(3):698–701.

87. Sundarakumar DK, Bhalla AS, Sharma R, Hari S, Guleria R, Khilnani GC. Multidetector CT evaluation of central airways stenoses: Comparison of virtual bronchoscopy, minimal-intensity projection, and multiplanar reformatted images. Indian Journal of Radiology and Imaging. 2011 Jul 30;21(03):191–4.

88. Hall SR, Allen CT, Merati AL, Mayerhoff RM. Evaluating the utility of serological testing in laryngotracheal stenosis. Laryngoscope. 2017 Jun;127(6):1408–12.

89. Luccichenti G, Cademartiri F, Fecci L, Carbognani P, Rusca M, Pavone P. Non-neoplastic tracheal lesions: Comparison between virtual CT endoscopy and fiberoptic bronchoscopy. Radiol Med. 2003;106:147–53.

90. García-Buenrostro N, González-Palafox MA. Implicaciones quirúrgicas de la TAC 3d en el diagnóstico de la estenosis laringo-traqueal. Revista Mexicana de Cirugía Pediátrica. 2005 Apr;12(2):65–80.

91. Lahjaouj M, El Bouhmadi K, Oukessou Y, Rouadi S, Abada R, Roubal M, et al. Laryngotracheal Stenosis: Clinical Aspects and Management Challenges. International Journal of Recent Surgical and Medical Sciences. 2021 May 10;07(01):017–22.

92. Sorantin E, Halmai C, Erdohelyi B, Palágyi K, Nyúl LG, Ollé K, et al. 3D cross section of the laryngotracheal tract. A new method for visualization and quantification of tracheal stenoses. Radiologe. 2003 Dec 1;43(12):1056–68.
